# Contralateral Masking in Bilateral Cochlear Implant Patients: A Model of Medial Olivocochlear Function Loss

**DOI:** 10.1371/journal.pone.0121591

**Published:** 2015-03-23

**Authors:** Justin M. Aronoff, Monica Padilla, Qian-Jie Fu, David M. Landsberger

**Affiliations:** 1 Department of Speech and Hearing Science, University of Illinois at Urbana-Champaign, Champaign, Illinois, United States of America; 2 Communication and Neuroscience Division, House Research Institute, Los Angeles, California, United States of America; 3 Department of Otolaryngology, New York University, New York, New York, United States of America; 4 Department of Head and Neck Surgery, University of California Los Angeles, Los Angeles, California, United States of America; University of Salamanca- Institute for Neuroscience of Castille and Leon and Medical School, SPAIN

## Abstract

Contralateral masking is the phenomenon where a masker presented to one ear affects the ability to detect a signal in the opposite ear. For normal hearing listeners, contralateral masking results in masking patterns that are both sharper and dramatically smaller in magnitude than ipsilateral masking. The goal of this study was to investigate whether medial olivocochlear (MOC) efferents are needed for the sharpness and relatively small magnitude of the contralateral masking function. To do this, bilateral cochlear implant patients were tested because, by directly stimulating the auditory nerve, cochlear implants circumvent the effects of the MOC efferents. The results indicated that, as with normal hearing listeners, the contralateral masking function was sharper than the ipsilateral masking function. However, although there was a reduction in the magnitude of the contralateral masking function compared to the ipsilateral masking function, it was relatively modest. This is in sharp contrast to the results of normal hearing listeners where the magnitude of the contralateral masking function is greatly reduced. These results suggest that MOC function may not play a large role in the sharpness of the contralateral masking function but may play a considerable role in the magnitude of the contralateral masking function.

## Introduction

Presenting a masker to one ear can affect the ability to detect a target signal in the opposite ear. This phenomenon, which is known as contralateral masking, is indicative of an interaction in the binaural system. In the normally functioning auditory system, signals travel along two pathways: the ascending pathway that brings signals from the cochlea to the cortex, and the descending pathway that sends signals from the cortex back to the cochlea, modulating the activity of outer hair cells and spiral ganglion cells. For normal hearing listeners, contralateral masking results in a masking pattern that is sharper and has a smaller magnitude than the ipsilateral masking function [1]. Both of these characteristics may reflect the role of the ascending auditory pathway [2], the descending auditory pathway [3], or both.

Along the descending pathway, the cochlea is affected by the lateral olivocochlear system (LOC) and the medial olivocochlear system (MOC). Both LOC and MOC can affect perception through their connections to the cochlea [4,5,6]. LOC efferents project from the superior olivary complex to the spiral ganglion cells. MOC efferents project from the superior olivary complex to the outer hair cells, inhibiting ipsilateral and contralateral outer hair cell function [4,7,8]. Because the outer hair cell function affects the inner hair cell function by altering the movement of the basilar membrane [4], MOC effects are circumvented by cochlear implants, which directly stimulate the spiral ganglion cells. Given that cochlear implant (CI) patients show evidence of contralateral masking [9,10], contralateral masking must be possible in the absence of MOC efferents.

Comparing CI patients’ contralateral and ipsilateral masking functions can help determine the role of the MOC projections in the sharpness and magnitude of the contralateral masking function. If the contralateral masking function is sharper than the ipsilateral masking function, it would suggest that this sharpness does not require the MOC efferents. In contrast, if the contralateral and ipsilateral masking functions are equally sharp, given that MOC efferents affect both contralateral outer hair cells and ipsilateral outer hair cells (via double crossing) [4] it would suggest that the MOC pathway plays an important role in either the sharpness of the contralateral masking function or the broadness of the ipsilateral masking function. Similarly, if the magnitude of the contralateral masking function is similar to that of the ipsilateral masking function, it would suggest that, for normal hearing (NH) listeners, the reduction of the effect of the contralateral masker results from enhancement by the outer hair cells. In contrast, if the magnitude of the contralateral masking function is smaller than that of the ipsilateral masking function, it would suggest that the LOC, the ascending auditory pathway, and/or collateral connections in the descending pathway are reducing the effect of the contralateral masker. To investigate the role of MOC efferents in these aspects of contralateral masking, ipsilateral and contralateral masking were measured in bilateral CI patients and the sharpness and magnitude of those masking functions was compared.

## Methods

### Subjects

Six bilateral CI subjects participated in this study. All participants had Advanced Bionics CII or HiRes 90K implants. Subject details are provided in Table 1.

**Table 1:** Participant details.

Subject	Age	Gender	Hearing Loss Onset	Cause	Implant Experience	Implant Type	Strategy
C3	57	Female	29 years old (progressive)	Hereditary	7 years (L) / 4 years (R)	HiRes 90K (L and R)	HiRes-S w/Fidelity 120 (L and R)
C14	48	Male	4.5 months (congenital)	Maternal rubella	4 years (L) / 8 years (R)	HiRes 90K (L and R)	HiRes-P w/Fidelity 120 (L and R)
C20	75	Female	7 years old (profound by age 9)	Red measles High fever	12 years (L) / 4 years (R)	HiRes 90K (L) / CII (R)	HiRes-S w/Fidelity 120 (L and R)
C21	59	Male	18 years old (progressive)	Ear infection noise exposure	1.5 year (L) / 1 year (R)	HiRes 90K (L and R)	HiRes-S w/Fidelity 120 (L and R)
C22	57	Male	34 years old (sudden)	Auto-immune	6 years (L) / 11 years (R)	HiRes 90K (L) / CII (R)	HiRes-S w/Fidelity 120 (L and R)
C23	73	Female	4 years old (congenital)	Congenital	7 years (L) / 0.6 year (R)	HiRes 90K (L and R)	HiRes-S w/Fidelity 120 (L and R)

### Apparatus

The experiment was conducted using the Bionic Ear Data Collection System (BEDCS version 1.17) for one ear and HRStream (version 1.0.2) for the other ear. Both devices were controlled by the same computer. The two systems do not provide the ability to precisely synchronize stimulation across the two ears. The temporal precision of interaural stimulation was approximately 5 ms. Both BEDCS and HRStream were controlled with custom Matlab-based software.

### Electric stimulation

Stimulation consisted of biphasic monopolar pulses. These pulses had a phase duration of approximately 140 μs and a pulse rate of approximately 255 pulses per second. These parameters were chosen to allow for future comparison with current focused stimulation, which requires large phase durations and consequently low pulse rates (e.g., [11]).

### Current steering

Current steering describes stimulation where current is presented in-phase on two adjacent electrodes such that the electric fields from the two electrodes interact and create a peak of stimulation between the two electrodes. The position of the peak of stimulation between the two electrodes is determined by the relative current amplitudes presented on each electrode. The coefficient α is used to describe the proportion of the total current presented to the basal of the two electrodes. The current steered electrical stimulation pattern is known as a “virtual channel.” Previous research has demonstrated that subjects with Advanced Bionics implants can distinguish places of stimulation differing by 20% (i.e. α differences of 0.2) of the distance between electrodes (e.g., [12]). The pitch of a virtual channel is perceived to be between the pitches typically provided by each of the two component electrodes. The pitch can effectively be "steered" anywhere between the two component electrodes by adjusting the relative amplitudes of each of the component electrodes (e.g., [13,14]). Steering virtual channels between two electrodes is perceived to cause a continuous change in pitch [15,16]. Furthermore, the spread of excitation of a virtual channel is the same as the spread of excitation from one physical electrode [17,18]. Steering between two adjacent electrodes produces no change in loudness [13]. Therefore, using virtual channels, the place of stimulation is not limited to the physical electrodes but instead can be provided as if there were a physical electrode at any location physically between the most apical and most basal electrode on the array. In the present experiment, current steering was used to increase the spatial resolution of the ipsilateral and contralateral masking functions.

### Unmasked thresholds

Unmasked thresholds were measured with 20 ms pulse train probes using a modified Bekesy tracking method. Subjects were asked to press the space bar on the keyboard while they could hear a sound and release it otherwise. The step size for the first four tracking sequences was 1 dB and the step size for the final six tracking sequences was 0.5 dB. For the initial ascending tracking sequence, the stimulation level started below threshold and increased by one step size until the subject indicated that the probe was audible. The probe amplitude was then increased by two steps sizes (ensuring audibility), and a descending tracking sequence began where the stimulation level decreased by one step size until the subject indicated that the probe was inaudible. At that point the probe amplitude was decreased by two step sizes (ensuring inaudibility) and an ascending tracking sequence ensued. A total of 10 tracking sequences were measured. Thresholds were calculated as the 20% trimmed mean of the final six sequences.

### Contralateral masked thresholds

Masked thresholds were measured using a simultaneous masking paradigm because contralateral masking has been shown to be greatest when the masker and probe are presented simultaneously [19]. The masker was a 500-ms pulse train presented at the most comfortable loudness level to the ear contralateral to the probe. As with the unmasked thresholds, the probe was a 20 ms pulse train. The probe was temporally embedded in the middle of the masker. The level of the probe was adjusted using the modified Bekesy protocol described above. Participants were tested separately with an apical and a middle masker. For most participants this was electrodes 3 and 9, respectively, although electrodes 6 and 9 were used for subject C23.

### Ipsilateral masked thresholds

Ipsilateral masked thresholds were measured after contralateral masked thresholds. Both ipsilateral and contralateral masking used the same ear for the probe. Except for subjects C3 and C14, the ipsilateral masker was placed at the peak (absolute maximum) of the contralateral masking function. Thus, if a masker on electrode 9 in the right ear produced the most masking at electrode 6 on the left ear, the ipsilateral masker was placed on electrode 6 on the left ear. For subjects C3 and C14 the masker was shifted from the peak by 1.2 electrodes or less, because peaks were calculated based on Gaussian fits rather than the absolute peak.

The contralateral and ipsilateral maskers were loudness balanced using a double staircase adaptive protocol. The step size for the first four reversals was 1 dB. The step size for the next six reversals was 0.5 dB. (smaller step sizes were used for some subjects to avoid presenting stimuli above acceptable maximum loudness levels). The last six reversals were averaged to obtain a threshold. Ipsilateral masking was measured with the same procedure used for contralateral masking. The pulse train for the probe was temporally embedded in the middle of the masker. The pulses for the masker and probe were temporally interleaved.

### Ethics statement

All participants provided written consent. The procedures were approved by the St. Vincent Medical Center institutional review board.

## Results

Data are available in S1 Dataset. Bootstrap analyses were used to minimize the potential effects of non-normality in the data. Bootstrap analyses avoid the assumption of normality by conducting tests on distributions based on the original data set rather than on normal distributions that may not accurately reflect the data. Trimmed means, which are a cross between a mean and a median, were also used to minimize the potential effects of outliers in the data. For more details, see [20,21] and the Appendix in [22].

**Fig 1:**
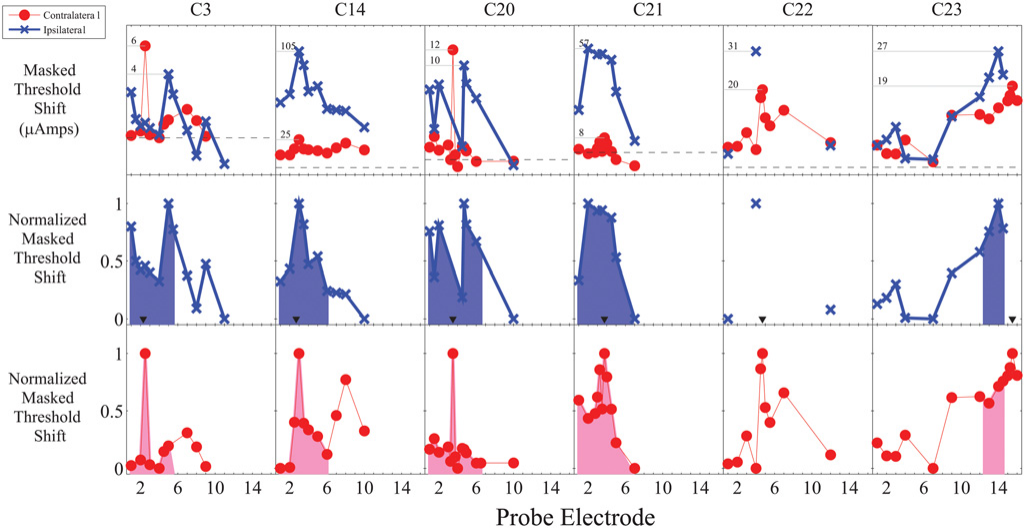
Raw and normalized masking functions for apical maskers showing both a generally sharper contralateral masking function as well as an inconsistent relative reduction in the magnitude of the contralateral masking function. The horizontal grey lines in the top panel indicate the peak of masking and corresponding numbers indicate the magnitude of the masked threshold shifts in μAmps. The black triangles in the middle panel indicate the masker location for ipsilateral masking. The shaded region in the bottom two panels is the area under the normalized masking function ± three electrodes (approximately ±3.3 mm) from the peak of masking.

**Fig 2:**
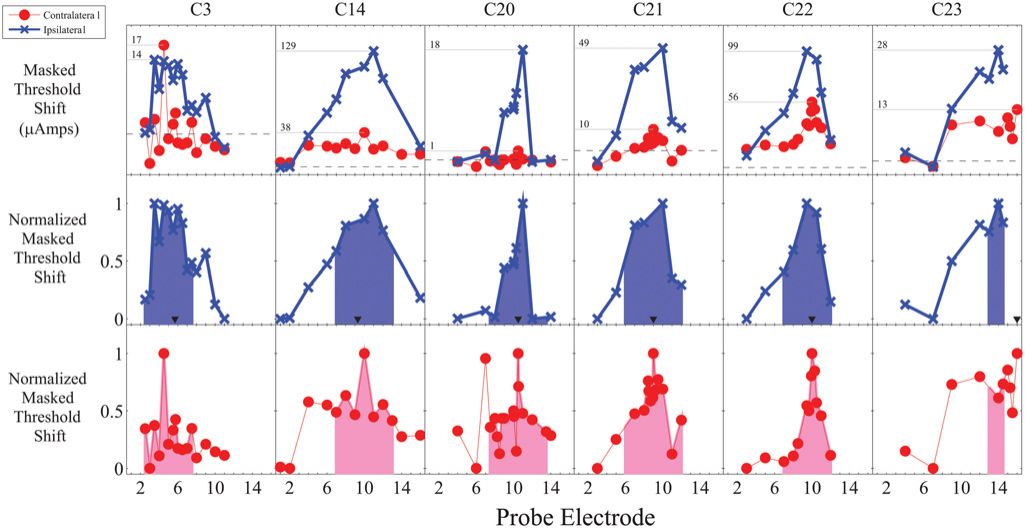
Raw and normalized masking functions for middle maskers showing both a generally sharper contralateral masking function as well as an inconsistent relative reduction in the magnitude of the contralateral masking function. The horizontal grey lines in the top panel indicate the peak of masking and corresponding numbers indicate the magnitude of the masked threshold shifts in μAmps. The black triangles in the middle panel indicate the masker location for ipsilateral masking. The shaded region in the bottom two panels is the area under the normalized masking function ± three electrodes (approximately ±3.3 mm) from the peak of masking.

For each participant and masking location, contralateral and ipsilateral masked threshold functions were calculated by subtracting the unmasked thresholds from the masked thresholds. The ipsilateral and contralateral masked threshold functions were then separately normalized for the range of masking. The raw and normalized threshold functions are shown in Figs. 1 and 2.

To compare the width of the normalized ipsilateral and contralateral masking functions, the area under the masking function within ± 3 electrodes (approximately ± 3.3 mm) of the peak of the contralateral masking function was analyzed. For some participants, it was not possible to measure masking for the apical or basal portion of the masking function due to either limitations in the amount of current that could be comfortably used (typically for ipsilateral masking) or because it would require stimulation beyond the edge of the array. A linear interpolation was used between measured stimulation points. The analyzed region is shown by the shaded areas in Figs. 1 and 2.

The area under the normalized contralateral masking function was divided by the area under the ipsilateral masking function to calculate the difference in masking area for contralateral and ipsilateral maskers. The difference measure was pooled across masker locations and analyzed with a percentile bootstrap pairwise comparison with 20% trimmed means. To do this, a bootstrap distribution of the difference measure (area under the contralateral function divided by area under the ipsilateral function) was obtained by resampling with replacement from the original difference measure. 2000 bootstrap distributions were generated (each with the same number of data points as the original distribution) and the 20% trimmed mean was calculated for each bootstrap distribution. These 2000 20% trimmed means were then ordered from smallest to largest and the 95% confidence interval consisted of the 50^th^ smallest (2.5^th^ percentile) and the 1,950^th^ largest (97.5^th^ percentile) 20% trimmed mean. If the entire confidence interval was less than 1, this indicated that there was a significant reduction in the masking area for contralateral masking.

**Fig 3:**
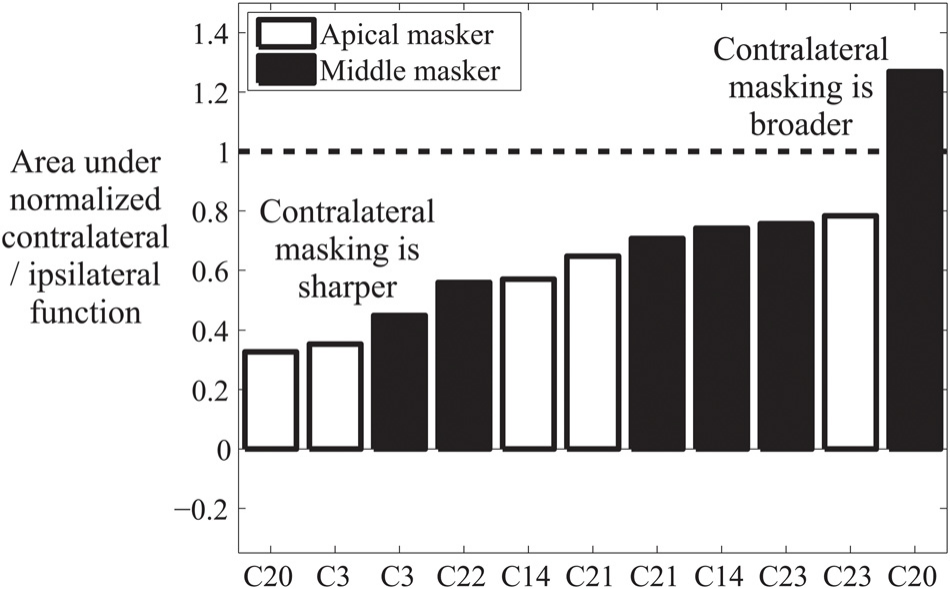
Results indicating that there is a consistent reduction in the spread of masking when the masker is moved to the opposite ear than the probe. Values below one (dashed line) indicate that there is a smaller area under the masking function when the masker and probe are in opposite ears than when they are in the same ear.

**Fig 4:**
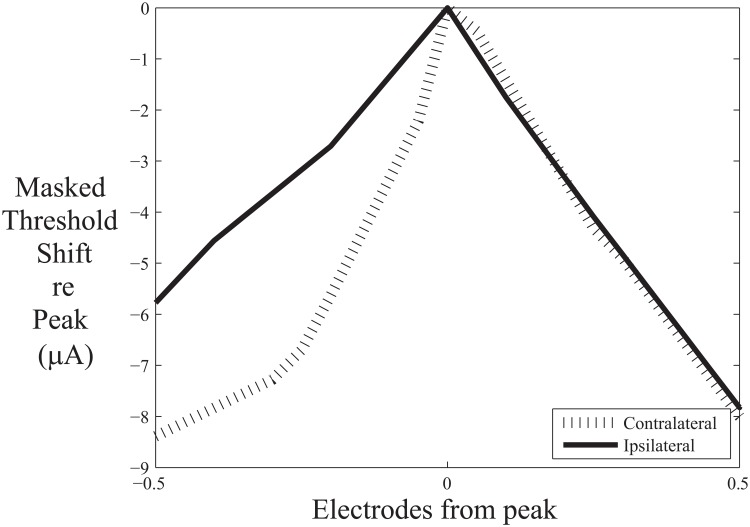
Results suggest greater reduction in masking at the apical end of the contralateral function. Masked threshold shift (in μA) is shown for the contralateral and ipsilateral masking functions relative to their respective peaks averaged across subjects and locations.

The results indicated that the area under the contralateral masking function was significantly less than the area under the ipsilateral masking function (95% confidence interval for the difference measure: 0.48 to 0.75; 20% trimmed mean = .65). As shown in Fig. 3, there was a reduction of the area under the masking function for contralateral masking for all subjects and masker locations except for the middle masker for subject C20. The reduction in area was asymmetric on average, with greater reduction at the apical end of the function (Fig. 4), although this result should be interpreted with caution given the considerable variability in the magnitude of masking across participants.

**Fig 5:**
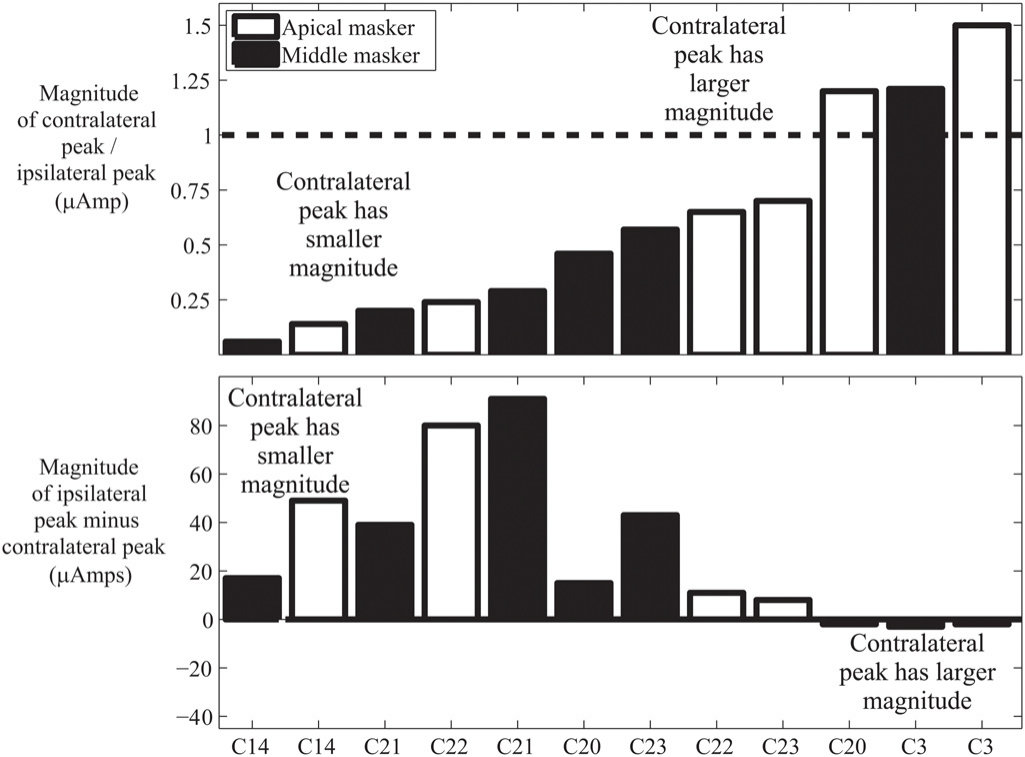
There was a significant but small and inconsistent reduction in the magnitude of the masking peak when the masker is moved to the opposite ear than the probe. The top panel shows the magnitude of the contralateral peak divided by the magnitude of the ipsilateral peak. Values below one (dashed line) indicate that the peak of masking is higher when the masker and probe are in the same ear. The bottom panel shows the magnitude of the ipsilateral peak minus the magnitude of the contralateral peak. Positive scores indicate that the magnitude of peak masking is lower when the masker and probe are in opposite ears than when they are in the same ear.

To determine if contralateral masking was smaller in magnitude than ipsilateral masking, the magnitude of the contralateral masking peak divided by the magnitude of the ipsilateral masking peak was analyzed using a percentile bootstrap pairwise comparison with 20% trimmed means. The results indicated that the contralateral masking peak was significantly smaller than the ipsilateral masking peak (95% confidence interval for the peak difference measure was .30 to .99; 20% trimmed mean = .52). The reduction in the magnitude of masking was not as consistent as the reduction in area with contralateral masking (see Fig. 5).

## Discussion

The results indicated that CI listeners’ contralateral masking functions are substantially sharper and moderately but significantly smaller in magnitude than their ipsilateral masking functions. Because signals traveling down the MOC pathway to the outer hair cells do not affect CI perception given the direct stimulation of spiral ganglion cells, these results suggest a role for either the LOC pathway, the ascending auditory pathway, or collateral connections in the descending pathway in the sharpness and magnitude of the masking function.

### Sharpness of the masking function

The sharpness of the contralateral masking function is similar to that found with NH listeners [1]. Additionally, Mills et al. [1] found that ipsilateral and contralateral masking slopes differed primarily on the apical side of the function, which is consistent with the CI data. These results indicate that the MOC pathway is not necessary for the sharpness of the contralateral masking function, although it is possible that the MOC pathway provides additional sharpening for NH listeners.

Previous studies with CI patients have generally found contralateral masking only for a small number of patients or when the data are averaged across individuals [9,10]. In contrast, in the current study, all patients demonstrated contralateral masking. The main reason for the difference between the current results and those of past CI studies likely reflects differences in techniques. The results of the current study indicate that the contralateral masking pattern can be exceedingly narrow, often extending less than the distance between electrodes. Previous studies have used probes spaced by one or more electrodes. In contrast, the current study used probes spaced apart by as little as 0.1 electrodes, implemented using current steering. The results suggest that such fine sampling may be required to adequately measure contralateral masking with CI patients.

Whether the contralateral masking function is sharpened as a result of the MOC efferents has particular importance for bilateral CI users. For CI patients, stimulation patterns within each ear may be quite different due to differences in electrode location and the distribution of healthy neurons [23,24,25]. Perceptually aligning stimulation in the two ears is critical for perceiving binaural cues [26,27,28]. Given the evidence that the sharpness of the contralateral masking function does not require MOC influence and thus also occurs with CI patients, considerable precision may be needed when perceptually aligning the two arrays, despite the broad spread of electrical current within each ear [29].

### Magnitude of the masking function

One of the striking differences between the CI and NH data is the difference in magnitude for contralateral and ipsilateral masking. With NH listeners, the magnitude of contralateral masking is typically a small fraction of that for ipsilateral masking [1,19]. However, some of the CI patients, such as C3 (apical and middle masker) and C20 (apical masker) have contralateral masking peaks that are larger than the corresponding ipsilateral masking peaks (see Fig. 5). This suggests that the reduction in contralateral masking magnitude for NH listeners likely reflects the role of the MOC pathway in enhancing attended frequency regions [4,8]. Alternatively, it may be the case that there is a decrease in ipsilateral masking for CI users rather than an increase in contralateral masking. With NH listeners, ipsilateral masking in part reflects two-tone suppression. However, given the role of the mechanical properties of the basilar membrane in two-tone suppression [30], this is unlikely to be a factor in masking for CI users.

The differences between NH and CI users may reflect differences between the nature of electric and acoustic stimulation. However, there appear to be similarities between the general shape and peak masking location with electric and acoustic maskers [9,10,19,31], although electrical stimulation appears to result in a broader masking pattern [9,32]. In contrast, differences between the two populations may reflect the underlying pathology that caused a patient to need a CI, or changes in the auditory system resulting from prolonged deafness and/or electrical stimulation.

Although the reduction in contralateral masking was not as great or consistent for CI users as it is for NH listeners, it was still significantly smaller than ipsilateral masking on average. That suggests that the MOC efferent connections to the outer hair cells are not the only route affecting the magnitude of contralateral masking and that the ascending pathway, the LOC pathway, and/or the collaterals from the descending pathway play a role in reducing contralateral masking.

In conclusion, the results from this study demonstrated that the MOC pathway is not necessary for the sharpness of the contralateral masking function or for a reduction in the peak of contralateral masking, although MOC function likely still plays a role in the reduction in the contralateral peak of masking.

## Supporting Information

S1 Dataset Complete dataset. (XLSX)
